# Gut-joint axis: Gut dysbiosis can contribute to the onset of rheumatoid arthritis *via* multiple pathways

**DOI:** 10.3389/fcimb.2023.1092118

**Published:** 2023-01-27

**Authors:** María del Socorro Romero-Figueroa, Ninfa Ramírez-Durán, Alvaro José Montiel-Jarquín, Gabriel Horta-Baas

**Affiliations:** ^1^ Centro de Investigación en Ciencias de la Salud, Universidad Anáhuac México, Mexico City, Mexico; ^2^ Laboratory of Medical and Environmental Microbiology, Department of Medicine, Autonomous University of the State of Mexico, Toluca, Mexico; ^3^ Dirección de Educación e Investigación en Salud, Hospital de Especialidades de Puebla, Instituto Mexicano del Seguro Social, Puebla, Mexico; ^4^ Rheumatology Service, Internal Medicine Department, Instituto Mexicano del Seguro Social, Merida, Mexico

**Keywords:** gut microflora, gut microbiome, rheumatoid arthritis, short-chain fatty acid, butyrate

## Abstract

Rheumatoid Arthritis (RA) is an autoimmune disease characterized by loss of immune tolerance and chronic inflammation. It is pathogenesis complex and includes interaction between genetic and environmental factors. Current evidence supports the hypothesis that gut dysbiosis may play the role of environmental triggers of arthritis in animals and humans. Progress in the understanding of the gut microbiome and RA. has been remarkable in the last decade. *In vitro* and *in vivo* experiments revealed that gut dysbiosis could shape the immune system and cause persistent immune inflammatory responses. Furthermore, gut dysbiosis could induce alterations in intestinal permeability, which have been found to predate arthritis onset. In contrast, metabolites derived from the intestinal microbiota have an immunomodulatory and anti-inflammatory effect. However, the precise underlying mechanisms by which gut dysbiosis induces the development of arthritis remain elusive. This review aimed to highlight the mechanisms by which gut dysbiosis could contribute to the pathogenesis of RA. The overall data showed that gut dysbiosis could contribute to RA pathogenesis by multiple pathways, including alterations in gut barrier function, molecular mimicry, gut dysbiosis influences the activation and the differentiation of innate and acquired immune cells, cross-talk between gut microbiota-derived metabolites and immune cells, and alterations in the microenvironment. The relative weight of each of these mechanisms in RA pathogenesis remains uncertain. Recent studies showed a substantial role for gut microbiota-derived metabolites pathway, especially butyrate, in the RA pathogenesis.

## Introduction

Although the precise etiopathogenesis of rheumatoid arthritis (RA) is not well understood, it is characterized by loss of immune tolerance and chronic inflammation (Zhang et al., 2015; Liu et al., 2016; Zhang et al., 2020; Li et al., 2022). The accumulation and activation of immune cells, including dendritic cells, macrophages, neutrophils, and T cell subsets, within the synovial tissue is a cardinal feature of RA (Buckley and McGettrick, 2018). Abnormalities in the immune response lead to dysregulated cytokine secretion and autoantibodies production. Anticitrullinated protein antibodies (ACPA) and rheumatoid factor (RF) are hallmark autoantibodies of RA (Burmester et al., 2014; Yue et al., 2019). The inflammatory environment produced by lymphocytes, macrophages, and fibroblast-like synoviocytes causes synovitis, leading to joint destruction (Opoku et al., 2022).

Although the trigger that leads to loss of immune tolerance is unknown, previous studies have shown that individuals at risk for RA showed IgA-ACPA before the onset of arthritis (Bos et al., 2014; Mankia and Emery, 2016; Yue et al., 2019). Therefore, a breach of tolerance at mucosal surfaces (lungs, gut, or oral mucosa) is considered an initial event in the pathogenesis of RA that can occur many years before disease onset. Experimental evidence has suggested that microbial factors may be possible initiators of autoimmunity (Moen et al., 2005). However, despite multiple efforts, it has not yet been possible to identify any microorganism causing RA.

Gut dysbiosis, an altered intestinal microbiota composition, has been implicated in the pathogenesis of multiple rheumatic diseases, such as RA, psoriatic arthritis, and axial spondyloarthritis (Gill et al., 2022; Wang et al., 2022b). The role of gut dysbiosis in the pathogenesis of RA has been widely studied from experimental animal models. Growing evidence has suggested the role of gut microbiota in the onset of arthritis. Studies in mice (Rosser et al., 2014; Liu et al., 2016; Jubair et al., 2018; Maeda and Takeda, 2019; Peng et al., 2019; Aa et al., 2020), rats (Huang et al., 2019; Peng et al., 2019; Yue et al., 2019; Xu et al., 2020; Xu et al., 2022a), and pigs (Mansson et al., 1971) consistently demonstrate that gut dysbiosis is associated with inflammatory arthritis development.

The germ-free condition has been found to alleviate arthritis symptoms in spontaneous mouse models of RA (K/BxN, SKG, and IL-1 receptor antagonist deficient mouse models) (Van de Wiele et al., 2016; Rogier et al., 2017). However, the introduction of segmented filamentous bacteria into germ-free mice caused the production of autoantibodies and arthritis (Ivanov et al., 2009). Furthermore, studies in rodents have shown that the intestinal microbial community undergoes marked changes in the pre-clinical immune-priming phase and precede the onset of inflammatory arthritis (Rogier et al., 2017; Jubair et al., 2018; Doonan et al., 2019; Zhang et al., 2019). In addition, differences in the gut microbiota before arthritis onset between collagen-induced arthritis (CIA)-susceptible and CIA-resistant mice are consistent with the view that bacteria can influence RA development (Liu et al., 2016).

Alteration of the gut microbiota *via* fecal microbiota transplantation (FMT) has been used to demonstrate the causal relationship between arthritis and microbiome composition. FMT from mice susceptible to CIA into germ-free mice increased the severity of arthritis. Similarly, the FMT enriched in *Prevotella copri* from RA patients exacerbates the arthritis of SKG mice (Maeda et al., 2016). In another study with mice, it was found that gut-induced dysbiosis by oral inoculation of *Porphyromonas gingivalis* exacerbated arthritis (Sato et al., 2017; Hamamoto et al., 2020). Conversely, it has been demonstrated that oral administration of *Prevotella histicola* in either preventive or therapeutic reduces arthritis severity (Marietta et al., 2016).

In a recent study, Chriswell et al. showed that *Subdoligranulum didolesgii*, a human gut commensal, triggers synovitis in the germ-free DBA/1 mice, along with deposition of complement and immunoglobulins (Chriswell et al., 2022). Significantly, mice monocolonized with *S. didolesgii* developed arthritis without an adjuvant trigger. Furthermore, serum transfer from arthritic mice into gnotobiotic mice injected intraperitoneally led to a rapid onset of arthritis.

Early administration of probiotics may be a potential strategy for moderating clinical arthritis. Treatment with *B. adolescentis* before arthritis can ameliorate inflammation through rebalancing immune responses and modulating the gut-associated responses such as gut microbiota, short-chain fatty acids (SCFAs), and gut permeability (tight-junction proteins) in the CIA mouse model (Fan et al., 2020a).

To date, limited studies have assessed the relationship between fungal gut microbiota, helminths, and RA. Lee et al. showed that intraperitoneal injections of a fungal cell wall component (zymosan or fungal β-glucan) into SKG mice in a specific pathogen-free induced autoimmune arthritis. In contrast, injections of an antifungal agent and antifungal cell wall component did not (Lee et al., 2022). In the CIA mice model (male DBA/1), gastrointestinal helminths (*Heligmosomoides polygyrus* and *Trichuris muris)* can protect against intestinal mucosa inflammatory conditions by modulating the gut microbiota and suppressing the inflammation associated with gut dysbiosis. The ability of helminths to relieve CIA has been attributed to their capacity to secrete molecules (ES-62) that exert immunoregulation and limit host pathology (Doonan et al., 2019).

These findings indicate that some gut bacteria species and fungi can induce arthritis in a genetically predisposed animal. Interestingly, significant changes in the fecal microbiota composition occur during pre-clinical and early onset arthritis stages of the CIA model. Therefore, gut dysbiosis plays a role in arthritis pathogenesis in various animal models of RA.

In recent years, studies have explored the association of gut microbiota with RA. Multiple studies have demonstrated that the gut microbiota composition on fecal samples differs between RA patients and healthy controls (HCs) (Scher et al., 2013; Zhang et al., 2015; Chen et al., 2016; Maeda et al., 2016; Pan et al., 2017; Yue et al., 2019; Kishikawa et al., 2020; He et al., 2022; Wang et al., 2022a). Despite discrepancies about the species involved, certain intestinal bacteria appear to be the link between gut dysbiosis and RA (**Table 1**). These findings suggested that gut bacteria can contribute to the pathogenesis of RA. In a recent meta-analysis, gut dysbiosis in RA patients was characterized by a depletion of anti-inflammatory butyrate-producing bacteria (i.e., Faecalibacterium) and enrichment of pro-inflammatory bacteria (i.e., Streptococcus) (Wang et al., 2022b).

**Table 1:** Summary of studies evaluating the role of Gut Microbiota in Rheumatoid Arthritis patients.

Autor	Country	Disease group	Case (n)	RF positive	ACPAs positive	Control group	Control (n)	Method	Overabundance	Lower abundance	Smoking	DMARDs	Diet
Alpizar-Rodríguez et al.	Switzerland	p-RA^1^	83	34%	46%	First-degree relatives	53	16sRNA	Prevotellaceae (*P.copri, P. stercorea, P. oralis, P. oulora, P. conceptionensis*) Lactobacillaceae	NA	19%	NA	NA
Rooney et al.	The UK	p-RA^2^	25	44%	100%	Healthy controls	44	16sRNA	Lachnospiraceae Helicobacteraceae Erysupelotrichaceae Bifidobacteriaceae	Bacteroidaceae Barnesiellaceae Methanobacteriaceae	60%	NA	History of vegetarian diet 12%
Scher et al.	EE.UU.	NORA e-RA	44 26	NORA: 95% e-RA: 81%	NORA: 100% e-RA 85%	Healthy controls	28	16sRNA WGS	NORA: Prevotellaceae (P. copri)	NORA Bacteroidaceae (genus Bacteroides) Lachnospiraceae Clostridiaceae	NA	NORA: Naïve 100% e-RA MTX 41% G.C.s 12% Biologic 12%	Patients with current extreme diet and probiotic use were excluded
Maeda et al.	Japan	NORA	17	82.4%	82.4%	Healthy controls	14	16sRNA	Prevotellaceae (*P. copri and P. stercorea*)	Bacteroidaceae (genus Bacteroides)	NA	Naïve 100% NSAIDs were allowed	Patients with extreme diet use were excluded
Zhang et al.	China	NORA e-RA	94 21	NA.	NA.	Unrelated healthy controls Healthy relatives	80 17	WGS MWAS	*Lactobacillus salivarius* *Bacteroides*, Gordonibacter pamelaear, *Eggerthella lenta*, *Clostridium asparagiforme*	Veillonella, *Hemophilus* ssp. *K. pneumoniae, Megamonas hypermegale, Sutterella wadsworthensis, Bifidobacterium bifidum*	NA	Naïve 82% Cs 18%	NA
Chen et al.	EE.UU.	e-RA	40	100%	83%	Healthy controls Healthy relatives	15 17	16sRNA	*Coriobacteriaceae (Eggerthella, Collinsella)*	Ruminococcaceae (F. prausnitzii)	NA	Cs 16.2% PDN 48.9% Biological 34%	NA
Kishikawa et al.	Japan	e-RA	82	74%	66%	Healthy controls	42	WGS	Prevotella spp. (*P. denticola, P. marshii, P. disiens, P. corporis* and *P. amnni*). *Gardnerella vaginalis.* *Bacteroides sartorii.*	NA	NA	Naïve 71% Cs 28% Biological 2%	Strict vegetarians were excluded
El Menofy et al.	Egypt	e-RA	45	NA	NA	Healthy	15	16sRNA	*Megasphaera, Adlercreutzia, Ruminococcus, Bacteroides, Collinsella*, and *Acidaminococcus*	*Acidaminococcus, Streptococcus, Gardenella, Anaerococcus*, and *Sphingomonas*	NA	DMARDs, GCs, and NSAIDs were allowed	NA
Ruiz-Limón et al.	Spain	e-RA	110	81.8%	80%	Healthy controls	110	16sRNA	*Collinsella* *Bifidobacterium*	*Oxalobacteraceae*	30%	DMARDs 100%. Biologic DMARDs 38.1%. GCs 18.2%.	Subjects with extreme diets or taking probiotics were excluded
Lee et al.	Korea	e-RA	99	82.8%	76.5%	Healthy controls	30	16sRNA	*Streptococcus* *Candida* spp.	*Bifidobacterium* *Blautia*	NA	csDMARDs 87.9% Biologics (40.4%)	NA
Chiang et al.	Taiwan	e-RA	138	75.8%	73.4%	Healthy controls	21	16sRNA	*A. muciniphila*		8.6%	csDMARDs Biologics (82.85) NSAIDs GCs	Subjects with extreme diets or taking probiotics were excluded
Jeong et al.	Korea	p-RA NORA	25	NA	NA	Healthy controls	25	16sRNA	Bacteroidales Prevotella (genus)	Erysipelotrichales Coriobacteriales Collinsella (genus)	NA	NSAIDs	Subjects taking probiotics or prebiotics were excluded

At the family level (at the species level)

16sRNA, 16S rRNA sequencing; Cs, conventional synthetic; DMARDs, Disease-Modifying anti-rheumatic drugs; e-RA, Established RA; MTX, methotrexate; MWAS, Metagenome-wide association study; NA, Information not available; WGS, Whole-genome shotgun sequencing; PDN, prednisone; p-RA, preclinical RA stage.

^1^Anticitrullinated protein autoantibodies (ACPAs) positivity and-or rheumatoid factor (RF) positivity or musculoskeletal symptoms with or without undifferentiated arthritis.

^2^Anti-CCP positive individuals with nonspecific musculoskeletal symptoms and without clinical evidence of synovitis.

Although the underlying mechanisms of the gut-joint axis still need to be investigated in more detail, new data suggest that gut microbiota is likely among the key players within the gut–joint axis. Gut dysbiosis precedes the onset of disease and could lead to changes in systemic immune responses, loss of tolerance, and the development of arthritis (Wang et al., 2022b). Notably, modifications in gut microbiota showed beneficial effects on symptom relief in animal models of RA, which were associated with the modulation of the immune response (Horta-Baas et al., 2021). However, the precise underlying mechanisms by which gut dysbiosis induces the development of arthritis remain unknown. This review aimed to highlight the mechanisms by which gut dysbiosis plays a role in the pathogenesis of RA.

## Mechanisms that account for the gut-joint axis in RA

**Figure 1:**
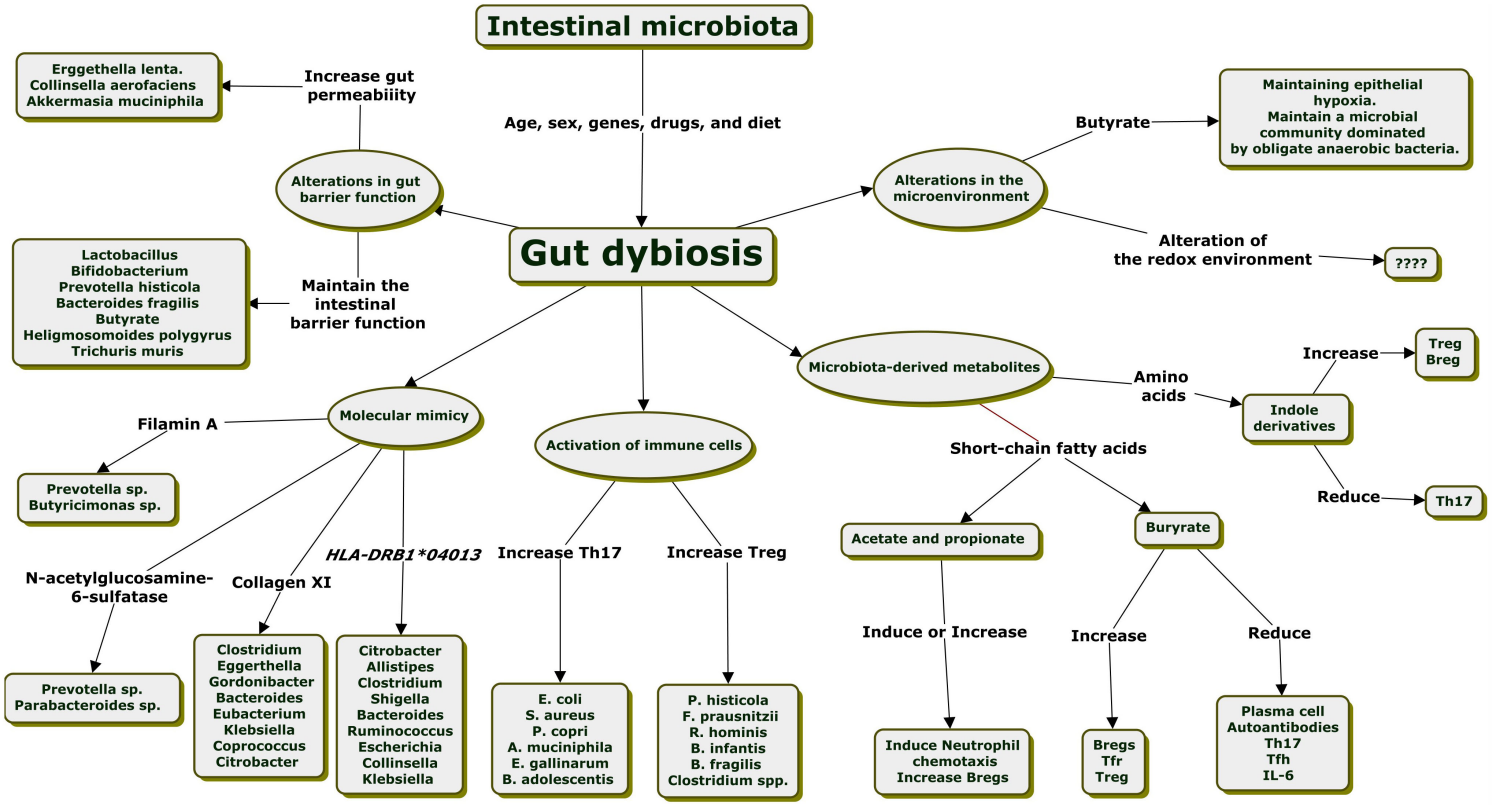
Mechanisms explained the relationship between intestinal dysbiosis and the development of rheumatoid arthritis.

Researchers have continued exploring the underlying mechanisms linking gut dysbiosis to RA in recent decades. Evidence suggests that gut dysbiosis can contribute to arthritis susceptibility through multiple pathways. Alterations in gut barrier function, molecular mimicry, alterations in the ratio of T helper 17 (Th17)/regulatory T (Treg) cells, an imbalance of T follicular helper cells (Tfh)/T follicular regulatory (Tfr) cells, cross-talk between microbiota-derived metabolites and immune cells, and alterations in the gut microenvironment are the mechanism proposed to explain a gut-joint axis through the interaction of gut microbiota with the host immune system (**Figure 1**) (Larsen, 2017; Picchianti-Diamanti et al., 2018; Zhang et al., 2020; Iljazovic et al., 2021a).

### Alterations in gut barrier function

Gut barrier function is part of the host’s defense against microorganisms, preventing pathogens from invading the intestine into the systemic circulation and extra-intestinal tissues and triggering immune responses (Correa-Oliveira et al., 2016; Xu et al., 2022b). The gut mucosal barrier, a monolayer of intestinal epithelial cells connect *via* tight junctions, separates the host from enormous amounts of antigens of both dietary and microbial origin. Mucus plays a vital part in this barrier by permitting access to host tissue for many diffusive molecules while limiting both the entry and colonization of microbes (Foster et al., 2017).

Zonulin is an enterotoxin secreted by enterocytes in response to dietary and microbial stimuli that disengages proteins zonula occludens-1 (ZO-1) and occludin from the tight junction (TJ) complex, leading to intestinal barrier damage, an increased permeability, translocation of bacterial products in the blood, and initiation of inflammatory responses (Xu et al., 2022b; Tajik et al., 2020; Audo et al., 2022). Zonulin induces T-cell-mediated mucosal inflammation and may control immune cells’ transmigration from the gut into the joints (Tajik et al., 2020).

The gut barrier is controlled by fine-tuned communications between gut microbiota and the host immune system (Litvak et al., 2018). Luminal antigen sampling by enterocytes *via* the transcellular pathway and dendritic cells regulates molecular trafficking between the intestinal lumen and the submucosa, leading to either tolerance or immune response to non-self. The loss of mucosal barrier function affects bacterial and antigen trafficking and allows microbes and their products to cross into the lamina propria and sub-epithelial spaces. The interaction of Toll-like receptors (TLR) and pathogen-associated molecular patterns (PAMP) on microbes have the potential to activate the immune system, leading to the production of pro-inflammatory cytokines such as interleukin (IL)-6 (IL-6), tumor necrosis factor-alpha (TNF-α), or IL-1β to eliminate the pathogen (Chiang et al., 2019; Opoku et al., 2022; Parantainen et al., 2022).

**Figure 2:**
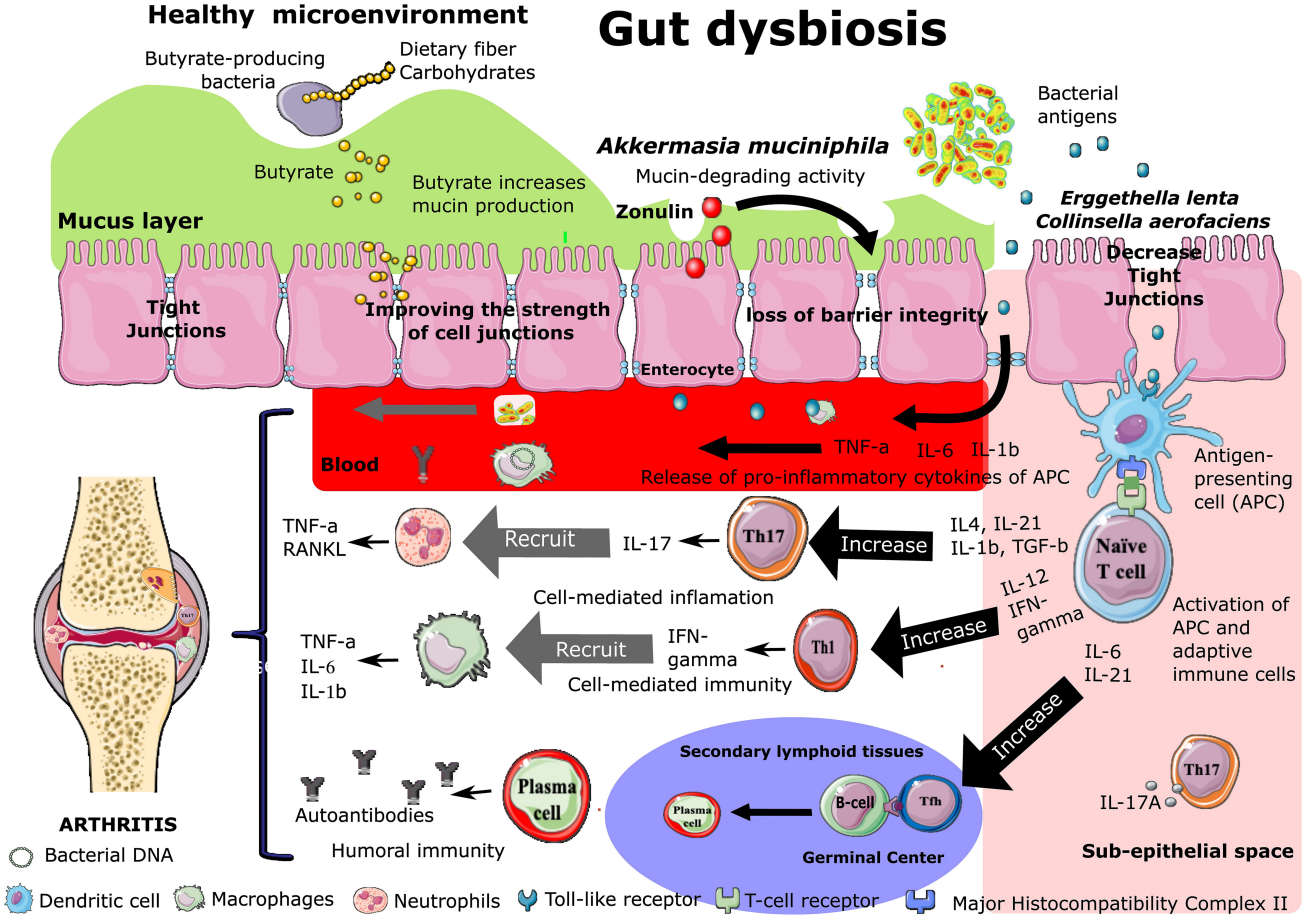
Effect of intestinal dysbiosis on intestinal permeability and B and T cell polarization during the development of rheumatoid arthritis. Increased Zonulin secretion is followed by increased intestinal permeability (“leaky gut”) associated with the disassembly of ZO-1 protein from the tight junction complex. Bacteria or their components are transported to the joints *via* secondary lymphoid organs or the bloodstream. After encountering the microbiota-derivated antigen presented by antigen-presenting cells (APCs), naive CD4+ T cells differentiate into various subsets, including at least Th1, Th17, and Tfh cells.

The disruption of the epithelial barrier function occurs in the pre-clinical phase of RA in murine models and humans (Tajik et al., 2020; Audo et al., 2022). Gut dysbiosis might trigger the breakdown of gut barrier integrity and the leakage of microbiota or their metabolites into gut tissue and even venous or lymphatic circulation, enabling exposure of the immune cells to bacterial antigens leading to local and systemic inflammation, increased pro-inflammatory cytokines such as TNF-α and IL-17A, and differentiation of autoreactive Th17 cells (**Figure 2**) (Berthelot et al., 2019; Chiang et al., 2019; Man et al., 2020; Tajik et al., 2020; Garabatos and Santamaria, 2022; Zhao et al., 2022). The migration of self-reactive cells to the joints can cause cartilage and bone damage (Zhao et al., 2022).

Translocation of gut bacteria (dead or alive) to joints from the intestine may lead to joint inflammation in RA patients. In agreement with these findings, some studies demonstrated that DNA from a variety of bacterial species (*Prevotella*, *Fusobacterium*, *Porphyromonas*, and *Bacteroides*) or bacterial cell wall constituents had been observed in serum and synovial fluids from RA patients (van der Heijden et al., 2000; Reichert et al., 2013; Zhang et al., 2015; Larsen, 2017; Zhao et al., 2018; Cheng et al., 2022). A recent study demonstrated that microbial invasion of the joint synovial fluid happens in the fourth stage of RA (RAS4) and that *Prevotella copri* was also found abundant in most synovial fluid samples of RA patients in RAS4 (Cheng et al., 2022).

It is still unclear how this bacterial nucleic acid (and perhaps still living bacteria from microbiota) reaches cartilage. Possible mechanisms, including bacteria or their components, are transported to the joints *via* the mesenteric lymphoid organs or the bloodstream. Another possibility includes that bacterial DNA is secondary to the migration of immune cells trafficking from the intestine harboring DNA (macrophages or leukocytes) (van der Heijden et al., 2000; Martinez-Martinez et al., 2009; Temoin et al., 2012; Berthelot et al., 2019; Cheng et al., 2022). Tajik et al. show that zonulin-dependent transmigration of immune cells from the gut into the joints occurs during the onset of arthritis. Furthermore, larazotide (a zonulin antagonist) treatment attenuated the enhanced intestinal permeability and blocked the migration of immune cells from the intestine to the joints (Tajik et al., 2020).

*Enterobacteriaceae* and *Klebsiella’s* lipopolysaccharide (LPS) could promote inflammation by increasing intestinal permeability (Chiang et al., 2019). The fiber-containing diet in mice colonized with *P. copri* increased inflammatory cytokine production, adaptive immunity activation, and gut barrier dysfunction (Jiang et al., 2022). In rodents, intestinal inflammation occurred earlier than the onset of arthritis, and restoration of the intestinal barrier by probiotics (*Bifidobacterium adolescentis*), butyrate, or using larazotide, was found to attenuate arthritis (Tajik et al., 2020; Fan et al., 2020a; Audo et al., 2022).

New-onset RA (NORA) patients had altered gut barrier integrity with lower expression of TJ proteins occludin and claudin-1 in intestinal epithelial cells on ileal mucosal and decreased ZO-1 in the colon, as well as increased serological gut permeability markers (i.e., zonulin/ZRPs, LBP and sCD14) (Tajik et al., 2020; Audo et al., 2022).

The gut microbiota produces metabolites that can maintain intestinal barrier function. Butyrate, a microbiota-derivated metabolite, contributed to the intestinal barrier function by multiple mechanisms. Butyrate increased the expression of the TJ protein claudin-1 and induced the redistribution of the TJ proteins occludin and ZO-1 in the cellular membrane (Tajik et al., 2020; Xu et al., 2022b). These protective effects seem largely concentration-dependent, with higher doses causing epithelial barrier disruption (Blaak et al., 2020).

Furthermore, butyrate is an essential regulator of intestinal barrier function through stimulation of mucin synthesis and quality (Blaak et al., 2020; Marazzato et al., 2022). Luminal-derived butyrate is a primary form of energy for the epithelial cells; 70% of the total amount of oxygen consumed by human colonocytes *in vitro* was used for butyrate oxidation (Correa-Oliveira et al., 2016; Van de Wiele et al., 2016; Kang et al., 2017; Lin and Zhang, 2017; Cani, 2018; Blaak et al., 2020; Fan et al., 2020b). These findings showed that butyrate is essential for a healthy colonic epithelium. Similarly, Indole-3-formaldehyde (3-IALD), a tryptophan metabolite, plays a role in maintaining intestinal epithelial barrier integrity and suppressing inflammatory responses dependent on AHR/IL-22 in mice (Xu et al., 2022b).

Some studies have investigated the mechanisms by which intestinal bacteria can alter the permeability of the intestinal barrier. Studies *in vitro* and in murine models of arthritis have demonstrated the arthrogenic role of *Collinsella*. *In vitro* experiments showed that the CACO-2 cell line cultured in the presence of *Collinsella aerofaciens* enhances gut permeability by decreasing the expression of tight junction protein ZO-1 in epithelial cells (Chen et al., 2016). In the humanized murine model of arthritis, DQ8 mice orally gavaged with *C. aerofaciens* showed an increase in gut permeability, and inoculation of *C. aerofaciens* into CIA-susceptible mice induces severe arthritis (Zhang et al., 2015; Chen et al., 2016). The overabundance of *Collinsella* in the gut microbiome has been reported in RA patients (Chen et al., 2016; El Menofy et al., 2022; Ruiz-Limon et al., 2022). In a recent study, *C. aerofaciens* was elevated exclusively in early RA (Cheng et al., 2022). Therefore, the overabundance of *C. aerofaciens* might contribute to the early breach in gut barrier integrity. In another study, the expansion of *Collinsella* was independently associated with inflammatory activity in RA patients (Ruiz-Limon et al., 2022). These findings suggest that the genus *Collinsella* seems to have an essential role in the pathogenesis of RA and its severity.

Similarly, the mucin-degrading activity of *Akkermansia muciniphila* can affect gut barrier function (Lin and Zhang, 2017). In the CIA mouse model, an overabundance of *A. muciniphila* was observed at the onset of arthritis (Peng et al., 2019). In one study, patients with active RA had a higher relative abundance of *Akkermansia* than those with inactive RA (Chiang et al., 2019). This effect may be related to its ability to degrade mucus and thus increase the exposure of resident immune cells to gut microbial antigens (Chiang et al., 2019). Furthermore, *Akkermansia* has also been associated with pro-inflammatory pathways, including the upregulation of B- and T-cell receptor signaling and the induction of M1-like macrophage response (Fan et al., 2021).

On the other hand, a probiotics-rich diet has been reported to ameliorate some RA symptoms by restoring barrier mechanisms in the gut mucosal (Opoku et al., 2022). Therapeutic administration of human gut-derived *Prevotella histicola* reduced the incidence and severity of CIA. *P. histicola* increased expression of ZO-1, preserving gut epithelium integrity in the context of inflammation (Marietta et al., 2016). *Lactobacillus* and *Bifidobacterium* could limit the development of autoimmune diseases in genetically susceptible individuals by increasing the expression of TJ proteins (Hills et al., 2019)*. Bacteroides fragilis* sphingolipids promote gut barrier integrity (Garabatos and Santamaria, 2022).

### Molecular mimicry

Gut bacteria can activate the immune system and trigger T-cell responses against self-antigens by molecular mimicry (Jethwa and Abraham, 2017; Zhang et al., 2020). The molecular mimicry or crossreactivity hypothesis proposes that an exogenous substance (i.e., a microbial agent with antigenic similarity to self-antigens) may trigger an immune response against self-antigens (Rashid and Ebringer, 2012). *Prevotella* contributes to arthritis development in mice by activating autoreactive T cells specific for the arthritis-relevant autoantigen Ribosomal Protein L23a (RPL23A) (Garabatos and Santamaria, 2022). Similarly, peptides derived from *Bacteroides fragilis*, *Candida albicans*, and *Streptococcus sanguis* are similar to collagen-type-II and induced cross-reactive responses in the CIA model (Costalonga et al., 2002; Yordanov et al., 2005; Zheng et al., 2020; Zhou et al., 2020).

Pianta et al., using discovery-based proteomics to detect HLA-DR-presented peptides in synovia or peripheral blood mononuclear cells, identified N-acetylglucosamine-6-sulfatase (GNS) and filamin A (FLNA) as targets of T and B cell responses in 52% and 56% of RA patients, respectively. GNS and FLNA were present in synovial fluid and inflamed synovial tissue (Pianta et al., 2017). The HLA-DR-presented GNS peptide has an evident homology with epitopes from *Prevotella* sp. (arylsulfatase protein) and *Parabacteroides* sp. (protein *N*-acetylgalactosamine-6-sulfatase). Similarly, the HLA-DR-presented FLNA peptide has homology with epitopes from proteins of *Prevotella* sp. (WP_028897633) and *Butyricimonas* sp. (WP_065219401.1). Therefore, sequence homology between T cell epitopes of two self-proteins and multiple gut microbial peptide epitopes may link gut microbiota and autoimmunity in RA.

Zhang et al. (Zhang et al., 2015) describe several gut microbial proteins as molecular mimicry for human self-antigens (collagen XI and *HLA-DR4/1*). Molecular mimicry of RA-associated antigens such as Collagen XI by gut microbial genes from *Clostridium, Eggerthella, Gordonibacter, Bacteroides, Eubacterium, Klebsiella, Coprococcus*, and *Citrobacter* was also suggested, with a number of the genes belonging to metagenomic linkage groups enriched in RA gut samples. RA-enriched genes from *Citrobacter, Allistipes, Clostridium, Shigella, Bacteroides, Ruminococcus, Escherichia, Collinsella*, and *Klebsiella* mimicked motifs in HLA-DRB1*04013.

Microbial antigens can be presented to CD4+ T cells by dendritic cells and macrophages, leading to the differentiation of inflammatory T cell subtypes. Therefore, molecular mimicry may partly explain the relationship between alterations in intestinal barrier function and the development of autoimmunity in RA patients.

### Gut dysbiosis induces the development of chronic inflammation and autoimmunity

The leading site of inflammation RA is the synovium, which includes a cellular surface layer of macrophages and fibroblast-like synoviocytes and an underlying tissue layer that contains fibroblasts, blood vessels, and lymphatics arrayed within a loose collagenous matrix. Immune cells ingress into the synovium is a critical process in the pathogenesis of RA (Qu et al., 2019). Pro-inflammatory cytokines and chemokines stimulate macrophages, neutrophils, T cells, and B cell infiltration (Block et al., 2016). Th1 and Th17 cells produce excessive pro-inflammatory cytokines, stimulating B cells to produce autoantibodies and macrophages to produce pro-inflammatory cytokines (Wang et al., 2019c; Fan et al., 2020a). These cytokines lead to synovial hyperplasia, pannus formation, and destruction of cartilage and joints. Pro-inflammatory cytokines induce fibroblasts to produce matrix metalloproteinases and RANKL (receptor activator of nuclear factor kB ligand), which mediate the destruction of bone and cartilage tissue, leading to the development of RA (Zhao et al., 2022).

Gut dysbiosis can lead to inflammation in the intestinal mucosa and tissue damage, promoting the loss of immune tolerance and the development of autoimmunity (Inda et al., 2019). Gut microbiota, primarily through microbiota-derived metabolites, has a role in regulating T cell functions and could disrupt gut immune homeostasis through abnormal antigen presentation and modulating the adaptive immunity, especially in the polarization of näive T cells to Th17 cells and generation of autoreactive B cells (Lin and Zhang, 2017; Wang et al., 2019b; Di Gangi et al., 2020; Zhang et al., 2020; Garabatos and Santamaria, 2022; Marazzato et al., 2022). Gut dysbiosis leads to inflammation by alterations in the ratio of Th17/Treg cells and an imbalance of Tfh/Tfr cells.

### Gut microbiota can modulate the Th17/Treg balance

Bacterial strains from the human intestine can regulate the differentiation and activation of Th17 and Treg cells (Narushima et al., 2014). Intestinal mucosa contains many Th17 and Treg cells (Zhao et al., 2022). Th17 cells usually are in the gut in a microbiota-dependent manner, maintaining tissue homeostasis and fighting against extracellular bacteria and fungi. Contrarily, intestinal Treg cells maintain immune tolerance to dietary antigens and gut microbiota, retain tolerance to self-antigens, and suppress the activation and proliferation of self-reactive effector T cells (Horta-Baas et al., 2017; Haase et al., 2018; Schinnerling et al., 2019; Sun et al., 2019). Microbiota-induced Tregs attenuate intestinal damage caused by exaggerated immune responses against pathogens (Kamada et al., 2013). Under physiological conditions, the functions of Th17 and Treg cells are in balance (Chiang et al., 2019).

Th17 mainly secrete IL-17 and IL-22 and intervenes in developing chronic immune-mediated inflammatory diseases, including RA (Feng et al., 2022). IL-17A is a potent inducer of matrix metalloproteinases, recruits neutrophils to the joint, and stimulates osteoclastogenesis resulting in cartilage and bone destruction (Amdekar et al., 2013; Pineda et al., 2014; Lee et al., 2015; du Teil Espina et al., 2019; Huang et al., 2019; Marazzato et al., 2022). Alteration in the ratio between Th17 and Treg cells plays a crucial role in the early phase of RA development (**Figure 2**) (Lee et al., 2015; Marazzato et al., 2022).

Gut-derived Th17 cells are thought to be essential in the link between gut microbiota and RA (Buckley and McGettrick, 2018). In animal models of arthritis, a pathogenic role of gut-derived Th17 cells has been demonstrated (**Table 2**). Microbiota from CIA-susceptible mice showed an altered ratio of Th17/Tregs cells, characterized by increased Th17 cells and reduced Treg cells (Pineda et al., 2014; Liu et al., 2016; Wu et al., 2018). Before the onset of CIA, Th17 cells aggregate in germinal centers. The release of autoantibodies and cytokines into circulation carries them to tissues and organs, leading to the activation of macrophages culminating in the release of pro-inflammatory cytokines (IL-6, IL-1, TNF-α, and IL-17). Germ-free mice conventionalized with the gut microbiota from CIA-susceptible mice, which have higher levels of serum IL-17, develop greater severity of arthritis (Liu et al., 2016). Maeda et al. demonstrated that FMT from RA patients in germ-free SKG mice could activate autoreactive T cells and an increased number of Th17 cells in the intestine compared with SKG mice inoculated with fecal microbiota of HCs (Maeda et al., 2016).

**Table 2:** Summary of possible underlying mechanisms by which intestinal dysbiosis contributes to the development of arthritis in rodents with collagen-induced arthritis.

	Model	Animal	Key findings
Marietta et al.	CIA	DBA/1 mice	Mice gavaged with *P. histicola* showed reduced IL-2, IL-17, TNF-a, and increased IL-4 and IL-10. Mice treated with *P. histicola* showed a reduction in anti-CII antibodies. Mice treated with *P. histicola* had increased numbers of CD103+ intestinal dendritic cells. *P. histicola* treated mice had a significantly lower gut permeability.
Hiu et al.	CIA	DBA/1J mice	The butyrate treatment alleviated arthritis severity. IL-1β, IL-6, and IL-17A were significantly downregulated in the butyrate group. In contrast, butyrate upregulated the mRNA expression level of IL-10 in synovial tissues. Butyrate promoted the polarization of Treg but not Th17 cells.
Xu et al.	CIA	Sprague–Dawley rats	Did not find a correlation between changes in gut bacteria and changes in amino acids metabolites (tryptophan, histidine, and phenylalanine) Gut dysbiosis was characterized by bacteria related to butyrate metabolism. Tripterygium glycosides could lead to a variation in metabolites in the tryptophan and phenylalanine pathways.
Jiang et al.	CIA	DBA/1J mice	P. copri was capable of activating the TLR4 pathway and producing LPS-induced inflammation. The fiber-containing diet-fed (FCD) mice displayed elevated levels of anti-collagen antibodies and more Th17 cells in the mesenteric lymph nodes.
Tajik et al.	CIA	DBA/1J mice	Intestinal inflammation and an increase in intestinal permeability precede the onset of arthritis. Th1 and Th17 cells accumulate in the intestine before arthritis onset. Butyrate levels drop before the onset of arthritis, Reducing intestinal barrier permeability attenuates arthritis.

On the contrary, some bacterial gut microbiota species exert an anti-inflammatory effect by stimulating Treg cells. Therapeutic administration (preventive or therapeutic approach) of human gut-derived *P. histicola* reduced the incidence and severity of CIA in HLA DQ8-transgenic mice by triggering the generation of IL-10-producing Treg cells, decreasing Th17 responses in the intestine and CD11c+CD103+ dendritic cells in the gut and the spleen (Marietta et al., 2016). Rats orally gavaged with *B. adolescentis* before immunization had significantly higher Tregs frequency and lower TNF-α than that in the late *B. adolescentis* treated group (Fan et al., 2020a).

In the mouse gut, colonic Treg induced by Clostridium bacteria are vital players in gut homeostasis and prevent colitis (Alameddine et al., 2019). In the human colon microbiota, Clostridium IV *Faecalibacterium prausnitzii* induces the formation of Treg cells *via* the activation of dendritic cells and causes the secretion of IL-10 by T cells (Garabatos and Santamaria, 2022; Wang et al., 2022b). Another gut bacteria, Bacteroides fragilis, *via* its carbohydrate antigen polysaccharide A (PSA), may promote the differentiation of Treg *in vitro* or mice through dendritic cell modulation. Furthermore, PSA stimulates Treg cells and suppresses Th17 cell responses through an IL-2-dependent mechanism (Horta-Baas et al., 2017).

In humans, gut bacteria have been shown to influence the polarization of T-cell subpopulations. At the phylum level, *Verrucomicrobiota* showed a positive correlation with the absolute number of Tregs, while *Firmicutes* showed a negative correlation with the total number of Th17 cells in RA patients (Wang et al., 2022a). Increased abundance of *Prevotella* and *Collinsella* in patients with RA are correlated with the production of Th17 cell cytokines. Bacterial species associated with increased Th17 or Treg are presented in **Figure 1** (Liu et al., 2016; Sun et al., 2019; Fan et al., 2020b; Garabatos and Santamaria, 2022; Yang et al., 2022). Human-derived *Clostridia* are potent inducers of Treg cells (Narushima et al., 2014). Bacterial strains belonging to Clostridia cluster IV and XIVa stimulate the secretion of transforming growth factor beta (TGF-β) by intestinal epithelial cells, promoting the expansion of Treg cells in the colonic lamina propria (Kamada et al., 2013; Lin and Zhang, 2017; Sun et al., 2019).

The Th17/Treg cells ratio is skewed in favor of Th17 cells in RA patients compared to controls. One study demonstrated an alteration in Th17/Treg balance, with higher Th17 levels and lower Treg levels in the peripheral blood, from early RA patients compared to HCs (Marazzato et al., 2022). Furthermore, RA patients present an impaired function of circulating Treg cells and an increase in Th17 cells in plasma and synovial fluid (Horta-Baas et al., 2017). Treg cells in RA patients show a decreased suppressive activity, which can be related to the potential of Treg cells to convert into Th1-like Treg cells, secreting interferon-gamma (INF-γ) as well as Th17-like Treg cells, secreting IL-17 (Haase et al., 2018; Fan et al., 2020b). In a recent study, Wang et al. found that the number of Tregs and Th17/Tregs ratio were negatively correlated with disease activity in RA patients (Wang et al., 2022a). In another study, Chiang et al. demonstrated a positive correlation between the abundance of the phylum Euryarchaeota with serum levels of IL-6 or IL-17A (Chiang et al., 2019). These results indicate a correlation between gut microbiota and RA disease activity.

### Gut microbiota can modulate the Tfh/Tfr balance

The production of antibodies occurs through B cells, which require Tfh cells for activation. B cells produce antibodies against extracellular pathogens and toxins. Antibodies are produced within germinal centers, regulated by interactions between B, Tfh, and Tfr cells (Ribeiro et al., 2022). Tfh cells are a CD4 T cell lineage that interacts with B cells to form germinal centers, promote differentiation into plasma cells, promote class-switching, somatic hypermutation, and the generation of high-affinity antigen-specific memory B cells and antibody-producing cells (Diamanti et al., 2016; Wang et al., 2019b; Zeng et al., 2022). Therefore, Tfh cells control initiation and the outcome of the germinal center B cell response. IL-6 and IL-21 can induce naive CD4+ T cells to differentiate into Tfh cells. Furthermore, IL-21 produced by Tfh cells is a factor that potently promotes B cell activation (Xie et al., 2019).

Microbial antigens can induce differentiating of B cells, with the help of Tfh cells, to plasma cells. Segmented filamentous bacteria are responsible for the induction of Tfh cells in Peyer’s patches (PP). Using the K/BxN mice model, Teng et al. demonstrated that PP Tfh cells were essential for segmented filamentous bacteria-induced arthritis despite producing auto-antibodies occurring in systemic lymphoid tissues, not PP. Consequently, gut microbiota can regulate arthritis development by driving the induction and gut Tfh cells migration to the systemic lymphoid tissues and inducing autoantibody production (Teng et al., 2016).

Excessive Tfh cell activity can lead to autoimmunity. The proper regulation of Tfh cell differentiation is essential for normal immune function and for preventing autoimmune disease. Tfr cells can suppress Tfh cell-mediated humoral immunity by downregulating the production of effector cytokines such as IL-4, IFN-γ, and IL-21, which are essential for B cell activation and class switch recombination (Wang et al., 2019b; Takahashi et al., 2020). Consequently, Tfr cells maintain tolerance during the B cell response. The increase of Tfh and decrease in the number of Tfr cells are associated with the growth of self-reactive B cells, which lead to the production of high levels of self-reactive autoantibodies (Xie et al., 2019; Wang et al., 2019c; Cao et al., 2020; Takahashi et al., 2020).

Self-reactive antibodies are present in approximately 70 to 80% of RA patients (**Table 1**). Some studies show that the imbalance of Tfh and Tfr cells may be involved in the association between intestinal dysbiosis and RA pathogenesis. Block et al. demonstrated that antibiotic treatment of IL-17-deficient mice inhibited arthritis, refuting the concept of a role for Th17 cells in gut-regulated K/BxN mice-induced arthritis. Instead, the authors proposed that the ability of the gut microbiota to regulate arthritis was dependent on Tfh cells (Block et al., 2016). In another study, Zeng et al. showed that Tfh and Tfr cells were increased in spleen germinal centers in the CIA mice model and their levels and functions returned to normal after the anti-TNF-a and anti-IL-1β treatment (Zeng et al., 2022). Although the Tfh/Tfr ratio did not change significantly, the relative enhancement of B cell function remained as the final result, which may be related to the relatively higher Tfh cell level.

Two studies in experimental models report a relationship between microbiota-derivated metabolite butyrate and alterations with Tfh/Tfr cells. Dietary butyrate supplementation conferred anti-inflammatory benefits in a CIA mice model (DBA/1). A butyrate-rich diet started on the first day of collagen immunization significantly lowers the overall incidence of arthritis and reduces the severity of joint inflammation. These effects were explained by rebalancing Tfh cells and Tregs and reducing antibody production (He et al., 2022). In another study, butyrate prevented arthritis development in the CIA and SKG mice model. However, butyrate does not prevent collagen antibody-induced arthritis (CAIA) or the development of CIA when butyrate begins after booster immunization. These findings suggested that butyrate suppresses the initial phase of Tfh cell-mediated autoimmune responses rather than the effector phase of arthritis development (Takahashi et al., 2020).

In RA patients, a reduced number of Tfr cells has been associated with the elevation of autoantibodies and disease severity (Takahashi et al., 2020). Wang et al. have shown that both circulating Tfh and Tfr cells were increased in RA patients compared with HCs. The percent Tfh cells positively correlated with the serum levels of serum RF, ACPA, and disease activity score in 28 joints (DAS28) index. Conversely, the Tfr/Tfh ratio was negatively correlated with the level of serum RF, ACPA, and DAS28 (Wang et al., 2019c). Similarly, Cao et al. found that peripheral blood Tfh cells were increased in RA patients, while the frequency of Tfr cells and the ratio of Tfr/Tfh were significantly decreased compared to HCs. Furthermore, the Tfr/Tfh ratio was positively correlated with RF and negatively correlated with the DAS28 index (Cao et al., 2020).

In another study, Ribeiro et al. reported that the frequency of circulating Tfh and Tfr cells was decreased in patients with RA and that the Tfr/Tfh ratio was similar to HCs (Ribeiro et al., 2022). These results show inconsistent results on the role of the Tfr/Tfh ratio in the pathogenesis of RA. Further studies are required to determine the role of alterations in Tfh and Tfr cells in RA’s pathogenesis and whether the gut microbiota modulates these cells during the development of arthritis.

### Cross-talk between microbiota-derived metabolites and immune cells

Recent works revealed that the relationship between gut dysbiosis and RA could be mediated by gut microbiota-derived metabolites (Yu et al., 2021; Yao et al., 2022). Microbiota-derived metabolites are critical for immune regulation (Haase et al., 2018; Iljazovic et al., 2021b; Yang et al., 2022). Gut dysbiosis may lead to alterations in fecal metabolites, and a deficiency of beneficial bacteria and their metabolites may stimulate the inflammatory response (Yu et al., 2021). Among gut bacterial metabolites, SCFAs, amino acids, and their metabolites have been implicated in the pathogenesis of RA (Chen et al., 2021).

### Short-chain fatty acids

SCFAs are small organic acids produced by intestinal bacteria through the fermentation of the cecum and colon’s undigested food components (mainly dietary fiber and carbohydrates) (Marazzato et al., 2022). SCFAs can regulate multiple metabolic pathways both in the gut and outside the intestine and are associated with a variety of physiological processes, such as energy balance, maintenance of the intestinal barrier, sugar/lipid metabolism, and immunomodulatory properties, thus contributing to disease prevention (Xu et al., 2022b).

The main SCFAs produced by intestinal bacteria in the human gut are acetate, propionate, and butyrate. Other SCFAs, include pentanoate, hexanoate, and heptylate (Jiang et al., 2022). Gram-negative bacteria, such as *Bacteroides*, primarily generate propionate and acetate, whereas gram-positive bacteria, such as *Firmicutes*, produce large amounts of butyrate (Lin and Zhang, 2017; Lee et al., 2019; Zhang and Frenette, 2019; Marazzato et al., 2022). Propionate and acetate are absorbed at the gut level and pass through bloodstream circulation, reaching and affecting distant tissues. Conversely, butyrate carries on its functions within the gut (Marazzato et al., 2022). The concentration of fecal SCFAs depends on dietary intake, the host’s gut microbiota community and host-microbiota metabolite flux, and the liver’s and small intestine’s absorptivity (Fan et al., 2020a).

The immunomodulatory properties of SCFAs are related to their effect on the innate and acquired immune system cells by inhibiting histone deacetylase (HDACs) (Correa-Oliveira et al., 2016; Lee et al., 2019). SCFAs could regulate neutrophils and macrophages and thus modulate the magnitude of inflammatory responses (Zhang and Frenette, 2019; Man et al., 2020; Wang et al., 2020). Acetate and propionate activate cell surface receptor GPR43 to induce neutrophil chemotaxis (Hills et al., 2019). Both *in vivo* and *in vitro* studies have demonstrated that SCFAs stimulate the polarization of M2 macrophages, which mainly exert an anti-inflammatory function (Jiao et al., 2020). At the level of intestinal macrophages, SCFAs cause down-regulation of the pro-inflammatory cytokine profile (Jiao et al., 2020). Furthermore, SCFAs play a role in colonic Treg cell homeostasis, reduced IgG, IgA, and IgE secretion, and plasma cell differentiation in human B cells in a dose-dependent manner (Xu et al., 2022b)

Neutrophils play essential roles in the pathogenesis of RA by promoting inflammation and facilitating autoantibody production (Cecchi et al., 2018; Zhang and Frenette, 2019; Aa et al., 2020). In RA, increased recruitment of neutrophils in synovial fluid occurs at the onset of this disorder (Zhang et al., 2015). Macrophages are one of the most abundant cell types in the synovium and are centrally involved in the pathogenesis of RA (Mondanelli et al., 2019; Yang et al., 2020). Activated synovial macrophages produce cytokines (IL-1β, IL-6, and TNF-α) that promote T-cell polarization and inflammation by activating a wide range of immune and non-immune cells (e.g., fibroblast and osteoclast) (Yang et al., 2020).

Results on differences between fecal SCFAs concentrations in RA patients compared to HCs demonstrate a reduced amount of SCFAs in samples of RA patients. In one study, levels of acetate, propionate, butyrate, and valerate were decreased in RA patients (Yao et al., 2022). In another study, early-RA patients presented significantly reduced propionate levels (Marazzato et al., 2022). Similarly, Takahashi et al. and Rosser et al. showed that the stool concentrations of butyrate were significantly lower in new-onset RA patients and inactive RA patients, respectively (Rosser et al., 2020; Takahashi et al., 2020). He et al. reported significant reductions in serum and stool butyrate levels in RA patients (He et al., 2022). Conversely, in Rosser et al. study, there was no difference in propionate or butyrate but a significant increase in acetate levels in serum samples of RA patients compared to HCs.

SCFAs play a role in colonic Treg cell homeostasis. Administration of SCFAs to mice with CIA can reduce the severity of arthritis by their ability to increase Foxp3+IL-10–producing Tregs (Smith et al., 2013). In another study, SCFAs positively correlated with Tregs and negatively correlated with pro-inflammatory cytokines (IL-17A, IL-6, TNF-a) in CIA rats (Fan et al., 2020a). In RA patients, the levels of acetate, propionate, and butyrate positively correlated with the frequency of B cells (Yao et al., 2022). SCFAs can diminish B cell differentiation and the production of autoantibodies (Piper et al., 2019; Yao et al., 2022). In addition, the production of SCFAs is one of the proposed mechanisms by which gut microbiota affects Treg cell differentiation (Yang et al., 2022). Colonization with *Clostridia* induces differentiation of peripheral Treg cells that have a critical role in suppressing inflammatory responses (Lin and Zhang, 2017).

### Butyrate

Butyrate is the most extensively investigated SCFAs (Hui et al., 2019). Butyrogenic bacteria are strictly anaerobic and oxygen-sensitive saccharolytic bacteria from the *Firmicutes* phylum. *Clostridia* clusters IV and XIVa, *Bacteroides fragilis, Ruminococcaceae*, and *Eubacterium* are the mainly intestinal bacteria producers of butyrate (Mizuno et al., 2017; Rogier et al., 2019; Takahashi et al., 2020; Wang et al., 2020).

Butyrate is critically involved in maintaining mucosal integrity and immune regulation (Guo et al., 2019; Lee et al., 2019; Garabatos and Santamaria, 2022; Wang et al., 2022b). The butyrate drives the metabolism of surface colonocytes toward mitochondrial beta-oxidation of fatty acids, which is essential for maintaining epithelial hypoxia (Litvak et al., 2018). Gut epithelial cells directly take up butyrate, and a lack of butyrate is associated with immune dysregulation in the intestine (Foster et al., 2017).

Butyrate has anti-inflammatory properties by regulating inflammatory gene expression and induction of Treg cells (Foster et al., 2017). Butyrate regulates pro-inflammatory cytokine expression (e.g., IL-1, IL-6, TNF-α), inhibits the expression of LPS-induced cytokines, inhibits LPS-mediated macrophage migration, modulates the function of dendritic cells (increased phagocytic activity and reduced T-cell stimulatory capacity), promoted conversion of naive T-cells into immunosuppressive Treg (Hui et al., 2019; Blaak et al., 2020; Wang et al., 2020). Butyrate suppresses pro-inflammatory effectors in lamina propria, macrophages, neutrophils, and differentiation of dendritic cells (DCs) from bone marrow stem cells *via* HDACs inhibition or suppressing the NF-kB activation (Correa-Oliveira et al., 2016; Koh et al., 2016; Wang et al., 2020).

There is mechanistic evidence for the effect of butyrate on mucosal immunity and inflammation, mainly from cell lines and animal models. *In vitro*, DCs treated with butyrate increase the expression of indoleamine 2,3-dioxygenase 1 and aldehyde dehydrogenase 1A2. These enzymes attenuate the immune activation through tryptophan depletion and the generation of retinoic acid, a molecule with immunosuppressive properties (Correa-Oliveira et al., 2016). Butyrate increased IL-10 and IL-23 production by macrophages and DCs (Correa-Oliveira et al., 2016; Mizuno et al., 2017; Wang et al., 2019a).

The effects of butyrate in relieving arthritis appear to occur indirectly by modulating the function of immune cells, especially Treg cells. In cell cultures, the treatment of butyrate on naïve T cells cultured under the Treg-cell-polarizing conditions promoted the IL-10 expression of Treg cells and further inhibited the pro-inflammatory cytokines secreted by Th17 cells (Hui et al., 2019).

In CIA, butyrate treatment attenuated arthritis onset, decreased serum zonulin concentrations, and reduced inflammation-mediated small intestinal shortening (Tajik et al., 2020). In the antigen-induced model of arthritis (AIA), in stool samples, there was a reduction of butyrate and acetate levels during the acute and remission phase of arthritis compared to pre-arthritic mice (Rosser et al., 2020).

The effects of supplementation with butyrate in the pathogenesis of RA have been evaluated in experimental mouse models. Dietary butyrate supplementation conferred anti-inflammatory benefits in a mouse model of arthritis by rebalancing Tfh cells and Tregs and reducing antibody production. He et al. compared a butyrate-rich diet (started on the first day of collagen immunization) to normal chow in the CIA model (He et al., 2022). The butyrate supplementation increased butyrate levels in stool and blood, accompanied by a significantly lower overall incidence of arthritis, reduced severity of joint inflammation, and milder arthritis. Dietary butyrate supplementation increased serum IL-10 levels and decreased serum IL-6 and autoantibodies. Butyrate increased the number of Tfr cells, especially in the draining lymph nodes, and reduced germinal center B cells. The anti-inflammatory benefits of butyrate in the DBA/1 mice model were explained by rebalancing Tfh cells and Tregs and reducing antibody production.

**Figure 3:**
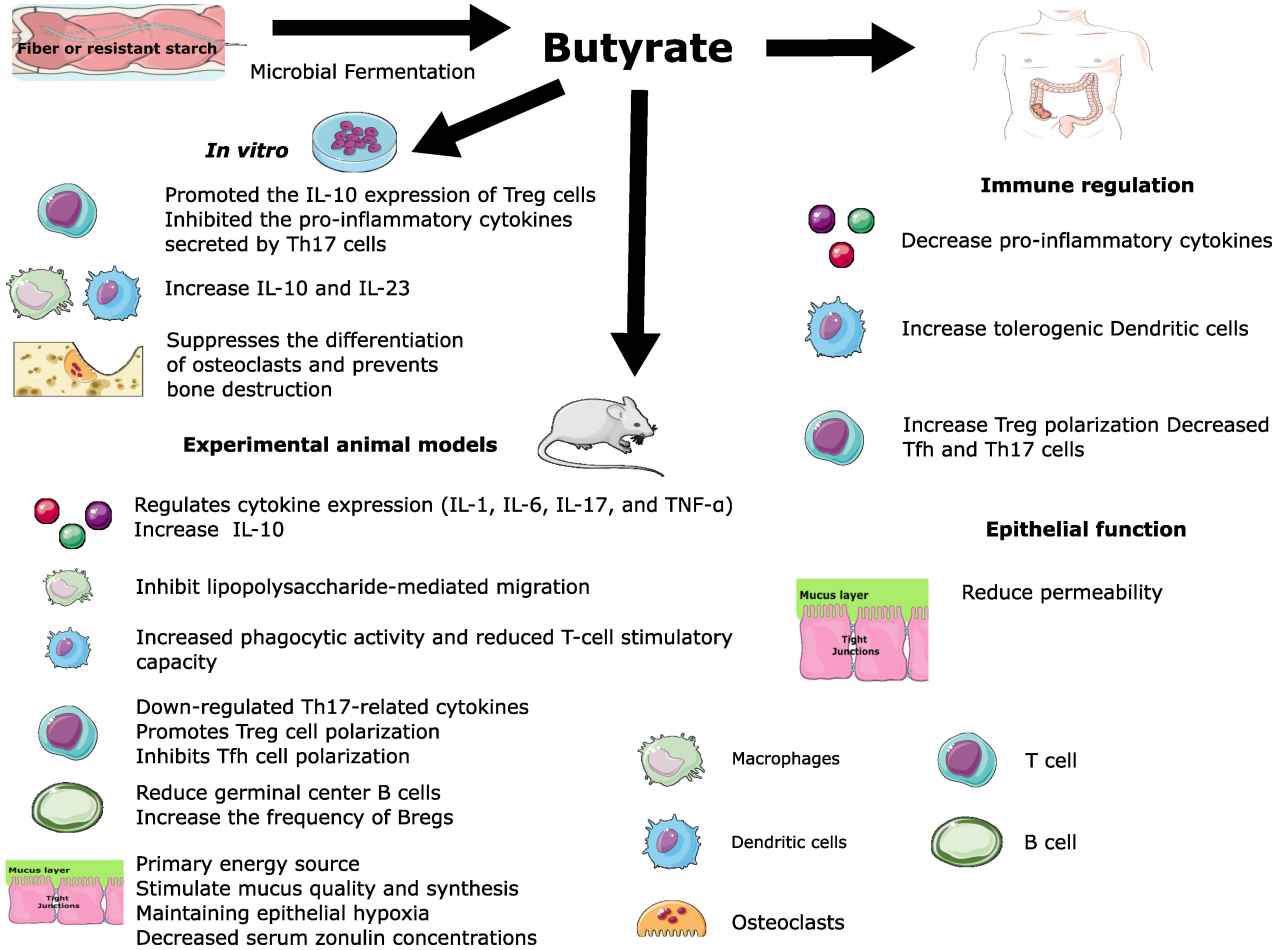
Pleiotropic immunomodulatory effect of butyrate.

Yao et al. demonstrated that supplementation of the three SCFAs before the onset of CIA in mice improved arthritic symptoms, increased the Bregs frequency, and decreased transitional B and follicular B cell frequency (Yao et al., 2022). These therapeutic effects were dependent on FFA2 receptors in CD19+ B cells. The fecal levels of acetate, propionate, and butyrate were positively correlated with the frequency of Bregs peripheral blood but not Tregs. Interestingly, treatment before the onset of CIA significantly improved joint inflammation and bone damage in mice, while administration after the start of CIA was less effective (Yao et al., 2022). Similarly, Rosser et al. reported that the supplementation with butyrate reduces the severity of arthritis in a Breg-dependent manner. The supplementation with butyrate before disease induction, but not acetate and propionate, reduced arthritis in Wild-Type mice compared to control mice (Rosser et al., 2020). However, butyrate supplementation failed to suppress disease in B-cell-deficient mice. These findings suggest that Bregs are necessary for the butyrate-mediated suppression of arthritis. Butyrate activates aryl-hydrocarbon receptor (AhR)-dependent gene transcription in B cells, supporting Breg function and inhibiting germinal center B cell and plasma cell differentiation. Nevertheless, butyrate no suppresses arthritis severity in Ahrfl/-Mb1cre/+, which has a B cell-specific deletion of AhR. Butyrate supplementation was associated with reduced TNF-α, IL-6, IL-17 production, and Th17 cell frequency. Interestingly, butyrate-mediated suppression was decreased in mice after Treg was depleted with an anti-CD25 depleting antibody treatment. Therefore, Treg also plays a role in mediating the suppression of arthritis by butyrate. These findings are consistent with the pleiotropic immunomodulatory effect of butyrate (**Figure 3**).

A recent study showed that the effect of microbial fermentation of fiber on host health could be context-dependent and species-dependent. Colonization of *P. copri* and a high-fiber diet led to the overproduction of organic acids, including fumarate, succinate, and SCFAs. Succinate promoted pro-inflammatory responses in macrophages. Furthermore, supplementation with succinate exacerbated arthritis in the CIA model. In patients with RA, succinate is abundantly present in synovial fluids, and these fluids elicit IL-1β release from macrophages (Jiang et al., 2022).

Gut dysbiosis in RA patients is characterized by a deficiency of butyrate-producing bacteria and an overwhelming number of butyrate bacteria consumers (He et al., 2022). In RA patients, higher butyrate levels were associated with increased Treg levels (Xu et al., 2022b). Patients with NORA displayed an increase in *Bacteroidetes* and a decrease in *Firmicutes*, *Proteobacteria*, and *Actinobacteria* compared to levels in HCs (Sun et al., 2019). It is possible that the reduction of *Firmicutes* can lead to inflammation in RA patients (Wang et al., 2022a).

In RA patients, total abundances of intestinal bacteria butyrate producers were lower in patients ACPA-positive compared to ACPA-negative patients. Conversely, butyrate consumers bacteria were higher in ACPA-positive than ACPA-negative patients. Furthermore, the increased abundance of butyrate-producing bacteria was associated with a lower incidence of deformed joint count and ACPA-positive, suggesting the potential roles of butyrate in alleviating inflammation. These anti-inflammatory effects may be attributed to increased Treg polarization, decreased Tfh and Th17 (but not Th1 or Th2) cell numbers, and a decrease in the production of pro-inflammatory cytokines. A higher proportion of circulating Treg was associated with high levels of stool butyrate (He et al., 2022).

### Amino acids

Metabolism of amino acids by intestinal bacteria may regulate inflammation and exert modulatory effects on the immune system (Mondanelli et al., 2019; Panfili et al., 2020; Yu et al., 2021). The cross-talk between amino acid metabolites and the immune cells has emerged as a possible mechanism by which gut dysbiosis could lean toward the development of inflammation or autoimmunity during the development of arthritis.

Microbiota-dependent tryptophan catabolites are abundantly produced within the intestine and are known to affect the maintenance of epithelial barrier function and immune homeostasis. The gut microbiota can also metabolize dietary tryptophan into indole derivatives. In host tissues, indole derivatives are known as ligands for the AhR, a ligand-activated transcription factor. AhR signaling contributes to immune homeostasis by modulating T cell differentiation. Indole derivatives are implicated in immune cell maturation and promote Treg differentiation while suppressing Th17 differentiation (Wang et al., 2019a; Yu et al., 2021; Hanlon et al., 2022; Yang et al., 2022). AhR expression and activation in DCs or T cells translate into Treg cell-mediated immunoregulatory effects, which dampen immune responses. However, in the presence of 6-formylindolo[3,2-b]carbazole, activation of AhR can promote the development of Th17 cells. Therefore, AhR plays a dual depending on the ligand nature, cell expression, and presence of other signals in the cell microenvironment (Panfili et al., 2020).

B cell-specific deletion of AhR in mice exacerbated arthritis, diminished IL-10 production by Bregs cells, and reduced the frequency of Tregs cells and expansion of inflammatory Th1 and Th17 cells compared with B cell AhR-sufficient mice (Piper et al., 2019). Rosser et al. demonstrated that butyrate reduced experimental arthritis severity *via* an increase in 5-hydroxy indole-3-acetic acid (5-HIAA), an indole derived from the decomposition of serotonin. The activation of AhR promoted the differentiation of B cells into Breg cells (Rosser et al., 2020).

Tryptophan metabolism would exert protective effects in experimental models of arthritis but not in all RA patients. RA patients may have reduced concentrations of tryptophan, 3-hydroxykynurenine (3-HK), and 3-hydroxyanthranilic acid (3-HAA), along with increased concentrations of kynurenine and xanthurenic acid, indicating that the kynurenine pathway is active in RA patients (Panfili et al., 2020). Few studies have evaluated the relationship between amino acid metabolites produced by gut microbiota and the pathogenesis of RA.

Recent work describes that the most highly enriched Kyoto Encyclopedia of Genes and Genomes (KEGG) pathway in RA patients was amino acid metabolism (e.g., alanine, aspartate, and glutamate) (Wang et al., 2022a). Two studies in Chinese RA patients reported that the amino acid pathways were significantly altered between the RA patients compared to HCs. Wang et al. (Wang et al., 2018) demonstrated decreased levels of tryptophan and glycine in RA patients compared to HCs; treatment with methotrexate returned amino acid levels to baseline. In another study by Yu et al., according to KEGG pathway enrichment analysis, the amino acid biosynthesis pathways were depleted in the RA group. These amino acids included L-arginine and ornithine, aromatic amino acids, and branched amino acids. Furthermore, RA patients exhibited lower levels of tryptophan metabolites in feces (Yu et al., 2021).

### Alterations in the gut microenvironment

The metabolic activity of the microbiota could also affect pathogen colonization. The butyrate influences the gut microbiota by driving the metabolism of surface colonocytes toward mitochondrial beta-oxidation of fatty acids, which is essential for maintaining epithelial hypoxia. The consequent epithelial hypoxia helps maintain a microbial community dominated by obligate anaerobic bacteria, which benefit from converting fiber into SCFAs (Litvak et al., 2018).

The metagenomic analysis from stool samples of RA patients demonstrates an altered redox environment (Scher et al., 2013; Zhang et al., 2015; Kishikawa et al., 2020). Iron transport-related genes were enriched in early RA patients (Jeong et al., 2019). Kishikawa et al. showed that the abundance of the R6FCZ7 gene, related to the redox reaction, was significantly decreased in the metagenome of RA patients compared to HCs (Kishikawa et al., 2020). The R6FCZ7 sequences were linked to *Bacteroides uniformis*, *Bacteroides rodentium*, Bacteroides fragilis, and *Bacteroides* spp. These findings have suggested that the redox function of the microbiome, especially the genus *Bacteroides*, may have an essential role in the pathology of RA (Kishikawa et al., 2020).

## Discussion

This review highlights the multiple mechanisms by which alterations in the gut microbiota contribute to the pathogenesis of RA. The relationship between gut dysbiosis and joint diseases, called the ‘gut–joint axis,’ has been suggested to be involved in the pathogenesis of arthritis, such as RA, Psoriatic Arthritis, and Spondyloarthritis. The association of gut dysbiosis with chronic inflammation and the fact that gut dysbiosis is essential to trigger arthritis in experimental mice models suggest a role of gut dysbiosis in the onset of RA. It has been hypothesized that the interactions between gut microbiota and host lead to mucosal inflammation and the breaking of immune tolerance (Chiang et al., 2019).

There is evidence that RA may be associated with changes in the composition of fecal bacterial communities. However, some studies have demonstrated the association between fungal microbiota, gastrointestinal helminths, and RA. Findings derived from animal models suggest that gut dysbiosis is related to the onset of RA, a stage in which activation of the autoimmune system occurs, leading to chronic inflammation (Burmester et al., 2014).

Growing evidence reveals the mechanisms underlying the link between gut microbiota, their metabolites, and cells (immune and non-immune) involved in RA pathogenesis. Gut dysbiosis affects the functions of the intestine and other organs, including joints. Consequently, persistent gut dysbiosis is associated with intestinal inflammation and increased Th17/Treg cell ratio. It can contribute to a break in immunological tolerance and tissue damage by various mechanisms, including translocation of bacteria across the gut barrier, T helper cell skewing, and crossreactivity with autoantigens. A possible hypothesis could be that gut dysbiosis trigger the migration of self-reactive B or T cells from intestinal sites to secondary lymphoid organs and arthritic joints. However, the mechanisms by which gut dysbiosis can contribute to RA onset are still incompletely understood and remains to be further elucidated.

Experimental animal models have been helpful in the understanding of the mechanism associated between gut dysbiosis and arthritis. Most studies have focused on the effects of a specific family or strain of bacteria or gut microbiota derivatives on the differentiation of Treg and Th17 cells. In contrast, other types of cells have been less well-studied (i.e., neutrophils, osteoclasts, or fibroblast-like synoviocytes).

Evidence suggests that gut dysbiosis is involved in the pathogenesis of RA, but to date, finding proof of causality is still a significant challenge in this field. This review showed the recent findings highlighting the complex regulatory networks between gut microbiota and the immune system. Gut microbiota diversity is easily altered by multiple factors such as drugs, diet, health status, hygiene, and surrounding environmental microorganisms. Furhermore, the inflammatory and metabolic pathways are complex networks context-dependent by various factors, including genetics, diet, cell status, and environmental factors (Yang et al., 2022). The mechanism of gut microbiota involvement in the occurrence and development of inflammatory diseases is very complex, and research on how intestinal metabolites and the host interact to affect diseases is a hot topic (Xu et al., 2022a). Further studies are needed to assess the impact of intestinal dysbiosis and gut microbiota-derived metabolites rather than specific bacterial species to understand the mechanisms involved in RA pathogenesis.

A more comprehensive understanding of the underlying mechanisms in the relationship between gut dysbiosis and RA will help to develop new treatment strategies. The study of gut microbiota-derivated metabolites is of great interest due to their therapeutic potential (He et al., 2022). The beneficial effects of butyrate obtained in animal studies warrant further investigation of its therapeutic potential in the form of butyrate-rich diets or by butyrate supplementation. Similarly, considerable evidence shows that alterations of the intestinal barrier are related to the onset of AR, and that gut dysbiosis could influence the inflammatory activity of RA patients through the regulation of gut permeability. Therefore, future studies may employ strategies to avoid a leaky gut (e.g., diet, SCFAs supplementation, or zonulin antagonists).

The authors hope this review’s results can provide a valuable resource for future research to advance our understanding of the possible underlying mechanism in the relationship between gut dysbiosis and RA.

## Author contributions

All authors listed have made a substantial, direct, and intellectual contribution to the work and approved it for publication.
